# Signaling Molecules Governing Pluripotency and Early Lineage Commitments in Human Pluripotent Stem Cells

**DOI:** 10.22074/cellj.2016.3915

**Published:** 2017-02-22

**Authors:** Ali Fathi, Shahram Eisa-Beygi, Hossein Baharvand

**Affiliations:** 1 Department of Stem Cells and Developmental Biology, Cell Science Research Center, Royan Institute for Stem Cell Biology and Technology, ACECR, Tehran, Iran; 2 Department of Developmental Biology, University of Science and Culture, ACECR, Tehran, Iran

**Keywords:** Differentiation, Stem Cell, Nodal

## Abstract

Signaling in pluripotent stem cells is a complex and dynamic process involving multiple mediators, finely tuned to balancing pluripotency and differentiation states. Characterizing and modifying the necessary signaling pathways to attain desired cell types is required for stem-cell applications in various fields of regenerative medicine. These signals may help enhance the differentiation potential of pluripotent cells towards each of the embryonic lineages and enable us to achieve pure *in vitro* cultures of various cell types. This review provides a timely synthesis of recent advances into how maintenance of pluripotency in hPSCs is regulated by extrinsic cues, such as the fibroblast growth factor (FGF) and ACTIVIN signaling pathways, their interplay with other signaling pathways, namely, wingless- type MMTV integration site family (WNT) and mammalian target of rapamycin (mTOR), and the pathways governing the determination of multiple lineages.

## Introduction

Human pluripotent stem cells (hPSCs), including human embryonic stem cells (hESCs) and human induced pluripotent stem cells (hiPSCs), exhibit capacity for self-renewal and differentiation to induce embryonic germ layers. The hPSCs are applicable in the context of embryological/developmental studies and for cell replacement strategies in clinical settings. The signaling pathways underpinning cell- fate specification in human development are similar to those in mammalian models. However, these processes may be context/tissue/species dependent despite being highly conserved. 

More than a decade after the generation of hPSCs, success in determining the mechanisms governing pluripotency and organ development have resulted in efficient differentiation of these cells to all the germ layers (mesendoderm, neural ectoderm, neural crest, epidermis, extra-embryonic cells). The *in vitro* characterization of hPSC has enabled developmental biologists and embryologists to delineate its function in the context of embryogenesis. Although the interaction among some intracellular molecules is not known, extrinsic factors could maintain pluripotency and govern differentiation *in vitro*. Hence, we have strived, in this review, to explain how diverse signaling mediators (and their inter-dependence) may regulate the pluripotency and differentiation states of hPSCs. 

### Molecular mediators contributing to the stemness of human pluripotent stem cells

#### Fibroblast growth factor signaling 

**Fig.1:**
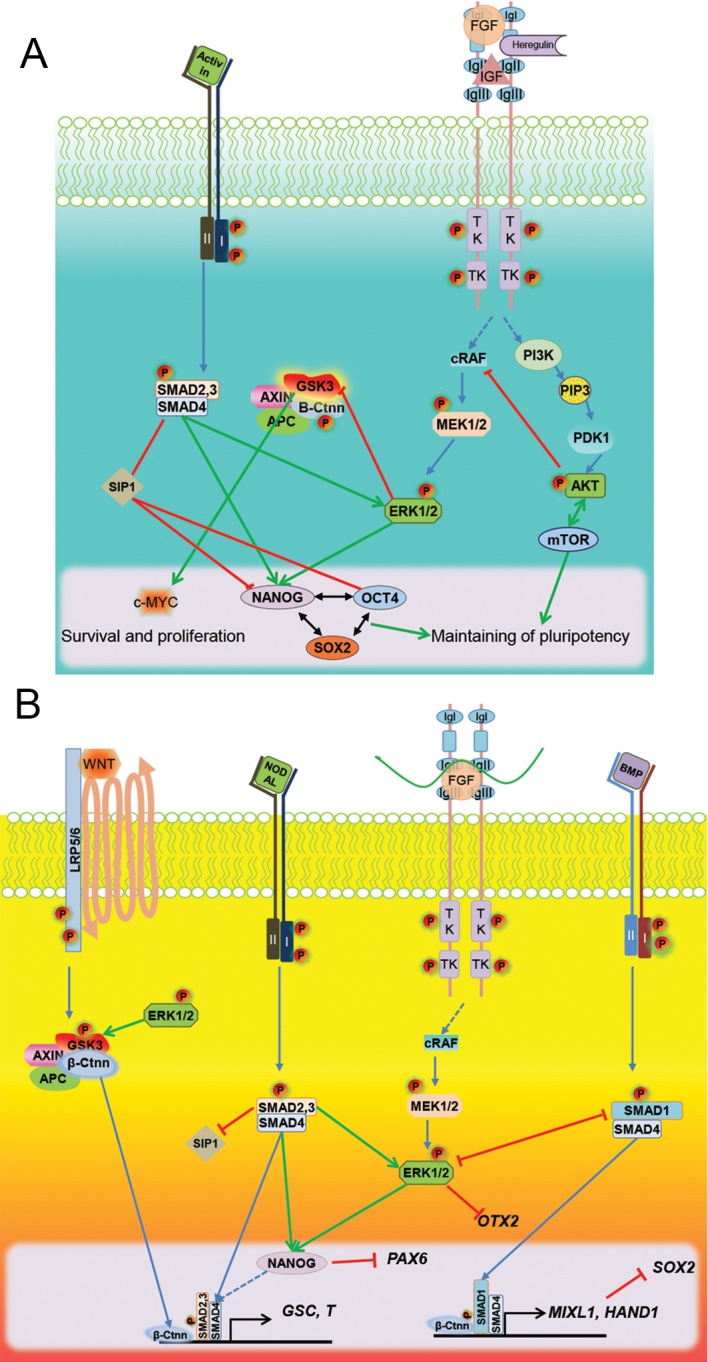
Molecular interplay of FGF and NODAL signaling in maintenance of pluripotecy in hPSCs and mesendodermal differentiation. A. Self-renewal of hESCs depend on activation of both fibroblast growth factor (FGF) and activin/nodal signals. Activin/Nodal signals bindsto typeI/II receptors. Hetrodimerization of receptors and their phosphorylation results in activation of R-Smads (SMAD2/3) and their binding to the co-SMAD (SMAD4). Internalization of the SMAD protein complex to the nucleus activates expression from NANOG directly. Indirectly, this could sustain the core pluripotency network and expressions of OCT4 and SOX2. SMAD proteins also inhibit expression of the SMAD interacting protein (SIP) which has a negative effect on OCT4 and NANOG expression, and could enhace neuroectodermal differentiation. FGF activation, by binding of their ligands [FGF, insulin-like growth factor (IGF) and heregulin] to tyrosine kinase receptors, results in phosphorylation and activation of phosphatidylinositol 3-kinase (PI3K) and cRAF. Activation of PI3K in turn activates AKT, which is involved in mediating inhibition of apoptosis and stimulation of cell proliferation, specifically via mTOR signaling in human pluripotent stem cells (hPSCs). Activation of cRAF activates mitogen-activated protein kinase MEK/ERK signaling that controls cellular processes such as survival and differentiation, and can maintain NANOG expression. A moderate signal of internal glycogen synthase kinase-3 (Gsk3) in hPSCs is needed for cell proliferation via c-Myc expression and B. Combined signals of mesendoderm specification. Activation of p-SMAD2/3 downstream of activin/nodal external signals reinforces the expression of NANOG and primitive streak specific genes (GSC and T). The high expression level of the ERK signaling, downstream of FGF, helps to stabilize th expression of β-ctnn and the mesendodermal genes. Phosphorylation of SMAD1 and its dimerization with SMAD4 occurs as a result of BMP binding to its receptors which enhance the expression of the posterior mesoderm and extra-embryonic mesodermal genes (Hand1 and Mixl1). Expressions of neural specific genes (*SIP1, PAX6, OTX2* and *SOX2*) that are inhibited by mesodermal signals are depicted by red lines.

Stem cells are defined by two unique characteristics, namely, the prowess for pluripotency and self- renewal, both of which are controlled by extrinsic environmental signals. One of the conserved pathways associated with hPSCs is mediated via fibroblast growth factor (FGF) signaling. Along with its downstream effectors, FGF activates genes that correspond to pluripotency (1). The binding of FGF ligand to the receptor tyrosine kinases initiates this pathway. This interaction increases the phosphorylation on tyrosine residues that lead to activation of the receptor’s intracellular kinase domain (2), resulting in the phosphorylation and activation of phosphatidylinositol 3-kinase (*PI3K*), and *cRAF*, to a lesser extent, which in turn activates the v-Akt (*AKT*). *AKT* mediates inhibition of apoptosis and stimulation of cell proliferation via mTOR signaling in hESCs (Fig .1A) (3). The activation of *PI3K* is required for hESC identity and its inhibition leads to onset of differentiation (4). FGF can be replaced by other extrinsic signals, such as insulin-like growth factor 1 (IGF1) and HEREGULIN, which can induce PI3K/ AKT signaling, suggesting that FGF signaling acts through PI3K/AKT in hESCs (1). Phosphorylation, with subsequent activation of *cRAF*, activates mitogen-activated protein kinase MEK/ERK signaling, which controls survival and differentiation. There is no evidence for the involvement of other FGF signaling mediators, such as p38, c-Jun N-terminal kinase (JNK), and protein kinase C (PKC), in hESCs. Inhibition of FGF signaling results in decline of *NANOG* homeobox (*NANOG*) expression in hESCs, but not in mouse epiblast stem cells, although the mechanism(s) remain unclear (5). Activation of ERK signaling antagonizes stem cell pluripotency and its phosphorylation can maintain *NANOG* expression through mesendoderm differentiation (6). Activation of AKT signaling could inhibit ERK signaling by binding to cRAF (7) and its repressive effect on ERK signaling was confirmed in hESCs (4). ERK signaling is also associated with mesoderm and neural differentiation and impairs pluripotency in hESCs (8,9). Therefore, blocking ERK phosphorylation is a valid strategy to inhibit the expression of genes driving pluripotency (Fig .1A). 

### ACTIVIN/NODAL signaling

The ACTIVIN/NODAL pathway is required for sustaining hESC pluripotency *in vitro*. Its cross-talk with the FGF signaling increases the expression of *NANOG*, octamer-binding transcription factor 4 (*OCT4*), and sex determining region Y (SRY)-box 2 (*SOX2*) genes (10,11). ACTIVIN and NODAL are members of the transforming growth factor beta (TGFβ) family proteins that bind to the same heteromeric type I (ALK4/5/7) and type II (ACTRIIB) receptors. Intracellular signals for ACTIVIN pathway include receptor regulated-Sma and MAD related protein 1 (SMAD) proteins (R-SMAD2/3), whose phosphorylation activates these proteins and causes dimerization with co-mediator-SMAD (Co-SMAD4). Upon translocation to the nucleus, these proteins bind SMAD-binding elements (SBE) on a locus in the genome upstream of other sites for transcription factors that control their target genes. Inhibitory SMADs (I-SMAD 6/7) can bind R-SMADs and facilitate their degradation by ubiquitin dependent proteolysis. ACTIVIN signaling is inhibited by the Cerberus, a BMP binding molecule, and left-right determination factor 2 (LEFTY2) proteins during neural plate formation, whereas NODAL is highly expressed in the inner cell mass of blastula and its expression is restricted to hESCs (11,12). Activation of *SMAD2/3* in hESCs directly activates NANOG expression by binding to the *NANOG* regulatory region (12,13) in combination with OCT4 and other regulatory proteins (14). Forced expression of *NANOG* could substitute ACTIVIN signaling and maintain pluripotency (13). Beyond activation of *NANOG* expression in hPSCs, *SMAD2/3* phosphorylation represses the expression of SMAD interacting protein 1 (*SIP1*), through enhancing pluripotency and mesendoderm differentiation. SIP1 protein is positively controlled by *SOX2* expression during neuroectoderm differentiation, and negatively regulated by *NANOG*, *OCT4* and *SMAD2/3* in hESCs (15). Knockdown of this gene is sufficient to maintain expression of the pluripotency genes in the absence of SMAD signaling. The hPSC pluripotency is mediated in part by the balance between neuroectoderm suppression by SMAD and the inhibition of mesendoderm differentiation by *SIP1* (Fig .1A). Although, it has been found that *TGFβ* inhibition by small molecules could enhance the derivation of mouse embryonic stem cells from blastula embryos and could maintain naïve pluripotency state despite the primed state in hESCs (16,17). 

### Glycogen synthase kinase 3 beta signaling

Glycogen synthase kinase-3 (GSK) is a conserved serine/threonine protein kinase downstream of numerous signaling molecules, including the WNT, FGF, epidermal growth factor (EGF), IGF and sonic hedgehog (SHH) pathways. Normally, the cytosolic GSK3 is part of a complex that is composed of the axis inhibitor (AXIN), adenomatous polyposis coli (APC), and beta-catenin (β-ctnn). It is involved in the phosphorylation and ubiquitin dependent proteolysis of β-ctnn. Upon phosphorylation, GSK3 becomes deactivated, followed by β-ctnn stabilization and translocation to the nucleus, where β-ctnn binds lymphoid enhancing factor/T-cell factor (LEF/TCF) and initiates the transcription of target genes (18). WNT, ERK and PI3K molecules are inhibitors for GSK3 and cause β-ctnn stabilization. However, in stem and dormant cells, GSK3 is active, as its activation is necessary for sustaining stem cell identity. There are discrepancies regarding the precise role of GSK3 inhibition and its activation for maintaining pluripotency in hESCs (4,19,20). 

Inconsistency regarding the function of GSK3 has emerged due to the phenotypic variability associated with various concentrations of GSK3-inhibitors. Although it is known that there are off-target effects of the GSK3 inhibitors (21), low levels of GSK3 inhibitors promote *c-MYC* expression and associate with cell self-renewal (22). Complete abrogation of GSK3 function (higher concentrations of the inhibitor) stabilize β-ctnn and direct hESCs toward a mesendoderm differentiation (4). The hESCs population heterogeneously expresses WNT signaling. Cells that highly express components of WNT signaling influence mesendoderm differentiation, whereas cells with low WNT signaling have remain in the pluripotent state and differentiate into neural ectoderm upon TGFβ inhibition (23). Taken together, β-catenin stabilization during differentiation of hPSCs, suggests a role for GSK3 activation in the hPSC ground state of pluripotency (Fig .1A). 

### Signaling cross-talk in mesendoderm differentiation of human pluripotent cells

The mesendoderm gives rise to the mesoderm and endoderm lineages during gastrulation. Although the developmental origin of bi-potent mesendoderm cells is unclear, it is assumed that these lineages diverged from a common precursor (24). The signaling molecules and instructor cells involved in ingression of the cells through the primitive streak and epithelial-mesenchymal transition (EMT) remain unknown. Evidence from vertebrates and hPSCs have demonstrated the crucial role of TGFβ in the emerging mesendoderm from epiblast cells (25). The Activin/NODAL/SMAD2/3 pathway exerts a direct impact on NANOG expression and the cross- activation effect on ERK phosphorylation is essential for mesendoderm induction. ERK phosphorylation downstream of the FGF signaling and SMAD2/3 prolongs NANOG expression, which, in combination with BMP signaling, induces the mesendoderm in hESC cultures (26). NANOG expression is a key element for fate decision between differentiation to mesendoderm and extra-embryonic tissues (6), which is directly controlled by SMAD2/3 and ERK pathways. NANOG expression is concomitant with *BRACHYURY*, a primitive streak gene, and its expression not only enhances expression of mesendodermal genes, but it can also hinder expression of the neuroectoderm gene, paired box 6 (*PAX6*) (12,27). Hence, *NANOG* expression, in conjunction with orthodenticle homeobox 2 (*OTX2*) and SIP1 inhibition by phosphorylated ERK and SMAD2/3 respectively, can block neural differentiation of hESCs (15). Lack of NANOG in hESCs cultures gives rise to the extra- embryonic lineage differentiation. Dimerization of SMAD2/3 with co-SMAD (SMAD4) and their internalization to the nucleus may activate mesendoderm gene expressions. However NANOG and the SMAD2/3 complexes have the same target genes during mesendoderm differentiation (6). 

BMP signaling is required for extra-embryonic differentiation and expression of the posterior primitive streak marker gene, mix paired-like homeobox (*MIXL1*) (20), whereas BMP inhibition increases the expression of anterior primitive streak, T, and goosecoid homeobox (GSC) in the presence of Activin signals and β-ctnn stabilization. Downstream phosphorylation of SMAD1 and its internalization with SMAD4 may activate the expression of the heart and neural crest-specific gene, *HAND1*, which is another determinant of mesodermal lineage (28). *HAND1* expression, downstream of BMP signaling, may abolish the expression of *SOX2*, and could blocking neuroectoderm differentiation (27). In contrast, ERK suppresses SMAD1 by MAPK mediated phosphorylation of the linker region of SMAD1. Intriguingly ERK inactivation leads to down- regulation of *NANOG* and activation of *HAND1* mediated autocrine BMP signaling (Fig .1B) (29). 

During anterior and posterior mesendoderm differentiation, the β-ctnn stabilization occurs without exogenous activation signals (20). 

Deprivation of the PI3K signal in hPSCs results in ERK activation downstream of SMA2/3 and FGF signaling; its activation blocks GSK3 and stabilizes β-ctnn, which synergistically with SMAD2/3, promotes expression of the mesendoderm genes (Fig .1B). Inhibition of SMAD2/3 suppresses the expression of the mesendoderm-specific genes, eomesodermin (*EOMES*) and *GSC*, even in the presence of β-ctnn stabilization, which confirms that both SMAD activation and WNT signaling are necessary for induction of mesendoderm in hPSCs cultures (4). 

### Emergence of the ectoderm 

#### Neural ectoderm: the default model 

**Fig.2:**
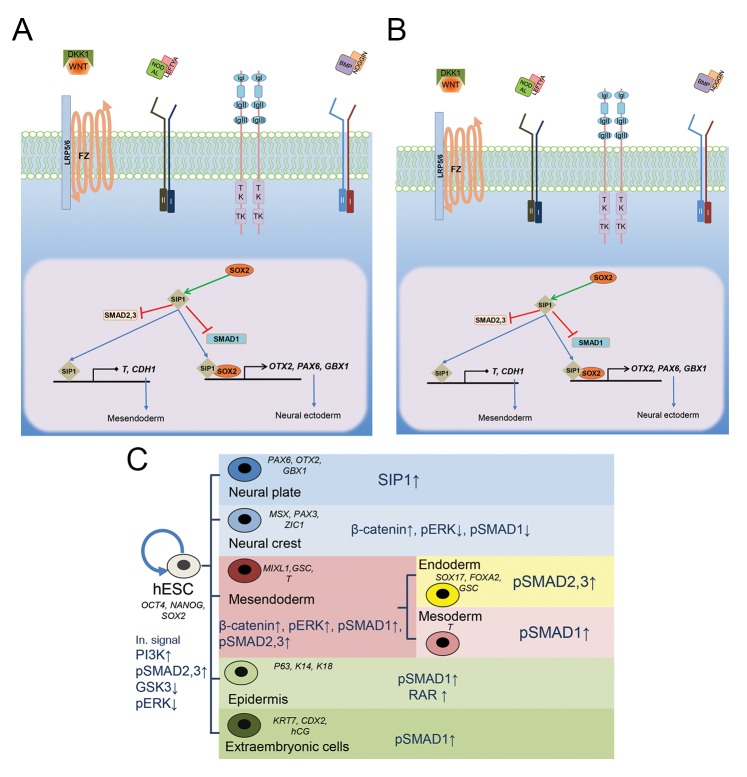
Neural ectoderm and neural crest specification in deprivation of the external signals. A. In the lack of external Activin/Nodal and ERK signals, the expression of *NANOG* decreases in favor of *SOX2* expression. *SOX2* expression, in turn, activates SMAD interacting protein 1 (SIP1) which blocks mesendoderm differentiation and actively promotes expression of neuroectoderm specific genes - *PAX6, OTX2* and *GBX1*, B. Neural crest (NC) determination occurs in two distinct steps that consist of neural plate border (NPB) and NC specification. NC specification occurs when the WNT molecule binds to its receptor Frizzled (FZ) and interacts with co-receptor LRP5/6, then these interactions can stabilizes β-ctnn by the canonical pathway. Activation of ERK by its phosphorylation downstream of the fibroblast growth factor (FGF) signals also contributes to glycogen synthase kinase-3 (GSK3) inhibition and indirectly assists with neural plate border determination by boosting the expression of the *MSX1, PAX3, PAX7* and *ZIC1* genes. Also, Leucine-rich repeats and immunoglobulin-like domains 3 (Lrig3) indirectly inhibit ERK activation is required for induction of the neural plate border. BMP signals cooperativly contribute to specify the NPB cells towards functional migratory NC cells by inducing expressions of the *SNAIL1, SOX9* and *FOXD3* genes, and C. Summarizing intracellular signals required for self-renewal and maintenance of hPSCs and its early differentiation. The major intracellular signals for hPSCs self-renewal and other differentiated progenies are depicted. High and low arrows indicate the amount of each signal inside the cells. Important transcribed genes are also shown for each step. For example, in the epidermis, the level of expression and phosphorylation of SMAD1 is high. This type of cell expresses the retinoic acid receptor (RAR), marked by p63, cytokeratin14 and cytokeratin18 gene expressions.

The processes involved in neural specification were initially described in xenopus during gastrulation. It was determined that the organizer or mesendoderm cells induced the neural axis (30) by expression of BMP antagonists, Chordin and Noggin . In the absence of TGFβ signals, the neural ectoderm emerges from the ectoderm, which suggests neural ectoderm specification through the default mechanism, without extrinsic factors (31). The notion of a default model for neuroectoderm induction (32) was supported by recent findings whereby dual SMAD inhibition, using BMP inhibitor (Noggin) and Nodal/Activin inhibitor (SB431542), was shown to be sufficient to induce neural induction in hESCs and iPSCs (33,34). The fate of epiblast cells towards neuroectoderm tissue is driven by the expression of Noggin, Lefty A (an extracellular antagonist of NODAL signaling) (35) and Dickkopf related protein-1 (DKK1), which antagonizes WNT signaling (Fig .2A) (20). The mechanisms through which intrinsic signals drive neuroectoderm transition from hESCs have been shown by introduction of SMAD interacting protein 1 (SIP1) (15). SIP1 is a zinc finger protein that interacts with SMAD1 and SMAD2/3 proteins (36) and acts as a repressor of the two mesendoderm specific genes, namely, *BRACHYURY* and *E-CADHERIN* (Fig .2A). Knockdown of SIP1 function in zebrafish induces neural patterning defects and reduction in neural crest precursors (37). Whereas expression of SIP1 leads to progression of neural ectoderm development, its expression is not necessary for neural induction (38). The precise mediators through which hPSCs regulate the balance of undifferentiated signals, along with determining when and how neural cell specifications occur and which signals trigger neurogenesis remain unknown. The default model of neuroectoderm induction, suggests a role for unbalancing of the pluripotency factors, *OCT4, NANOG* and *SOX2*, inside hPSCs (14). In the absence of external signals, expression is biased towards one of the trio of factors inside the epiblast cells. Both NODAL/ ACTIVIN and FGF signaling activate *NANOG* expression. In the absence of these signals, *NANOG* expression decreases in favor of *PAX6* and *OTX2* expressions (Fig .2A) (34). This also applies to BMP signaling, which positively controls *HAND1* and *MIXL1* expressions and mesendoderm specification (15). Lack of BMP activation results in decreased HAND1 and MIXL1 expressions and activates *SOX2* expression. Increased *SOX2* expression, along with lower expression of the other two factors (*OCT4* and, *NANOG*) induces neuroectoderm differentiation (5,39). SOX2 binds to the SIP1 promoter and activates its expression (15), this may be an inductive signal for neuroectoderm differentiation of hPSCs to facilitate expression of *OTX2, PAX6* and *GBX1* genes, following inhibition of mesendoderm specific *CDH1* and *T* gene expression (Fig .2A). 

### Fibroblast growth factor signaling and neural induction in human pluripotent cells

The extent to which FGF signaling pathway contributes to neuroectoderm specification of hPSCs is not well understood. The role of FGF signaling has been reported in neural induction for zebrafish (40), xenopus (41), chicken (9) and mice (8,9,31). However, FGF signaling contributes to neural patterning and its blockage leads to differentiation of hESCs towards peripheral neurons (27). Yet, whether FGF is necessary in hPSCs neural induction is contentious. Some studies have demonstrated that FGF acts as an instructor signal in neural commitment (42,43), whereas others have shown an inhibitory role for FGF signaling in this process (27,33). FGF signaling may affect neural induction through two mechanisms, namely, BMP-dependent and independent pathways. In the BMP-dependent pathway, mitogen activated protein kinase (MAPK), activated by FGF, phosphorylates the linker region of *SMAD1*, which marks SMAD for degradation by the proteasome system (29). Although this interaction has been confirmed in some vertebrates, there is no evidence for this process in hESCs (43). Other relevant pathways suggest ERK1/2 activation and downstream *SOX* gene expression, independent from BMP signaling (9). However, activation of ERK signaling can enhance neural specification through poly (ADP-ribose) polymerase-1 (*PARP-1*) mediated transcriptional regulation of the PAX6 gene expression in hESCs (42). Studies suggest that hPSCs resemble mouse epiblast stem cells and their state is primed in comparison to the naïve state of the mouse and chicken stem cells (44). A different hypothesis about the role of FGF in differentiation of hPSCs suggests that naïve stem cells, such as mESCs, require FGF to become primed for differentiation (lower potency in epiblast stem cells). The primed stem cells in hPSCs are a step-up to differentiation and do not require FGF for their neural induction. 

### Neural crest induction signals in human pluripotent cells

Neural crest (NC) cells are multipotent, migratory cells that transiently emerge in the border of the presumptive neural plate and epidermis during neural tube closure. These multipotent cells migrate during the embryo rostral-caudal axis and can differentiate into cranial mesenchymal cells (which make facial bone and cartilage), the peripheral nervous system, and melanocytes. They contribute to numerous other organs during organogenesis (45). Signaling molecules in NC specification come from nearby epidermis cells secreting WNT (46) and from underlying mesoderm that produce FGF signals (47). NC cells are also under a moderate concentration of TGFβ inhibitors from chordate mesoderm (48). The process of NC induction has a different timing compared to that of neural plate induction (49) and takes place in two interconnected steps (50). In the first step, ectodermal cells endure a range of WNT, FGF activating signals and TGFβ inhibitors to establish the neural plate border which expresses Msh homeobox 1,2 (MSX1,^2^), PAX3,7 and Zic family member 1 (ZIC1). In the second step, moderate BMP levels, along with WNT and FGF molecules, specify NC cells with expression of migratory signals, snail family zinc finger (SNAIL), SOX9 and forkhead box D3 (FOXD3) (Fig .2B) (51). Those signals have been successfully used for *in vitro* differentiation of hESCs into NC cells (52). WNT co-receptor, the low-density lipoprotein (LDL) receptor-related protein 5/6 (LRP5/6), are needed for robust activation of its canonical pathway (53) which stabilizes β-ctnn, facilitating its release from the GSK3 complex, and translocation to the nucleus. Activation of the FGF signal and its downstream ERK pathway can enhance the phosphorylation of GSK3 and β-ctnn to amplify the WNT signal for determining the neural plate border (Fig .2B). Background expression of BMP effectors, SMAD1/4, leucine-rich repeats and immunoglobulin-like domains 3 (*LRIG3*), which modulate FGF signaling, may be required for inducing faithful NC cells in the neural plate border (52). Apart from the requirement of WNT signaling for NC induction, other signaling pathways remain to be dissected and more mechanistic information regarding NC derivation from ectodermal cells, along with their sub-specification into peripheral neurons, Schwann cells, melanocytes and other cell types, is needed. 

### Epidermis

In the neurula stage, the definitive ectoderm divides into the neural ectoderm and surface ectoderm (epidermis). The surface ectoderm differentiates into the epidermis, which covers the entire embryo and consists of keratinocytes, nails, hair, sebaceous glands, olfactory and mouth epithelium, and the lens of the eyes. In vertebrates, a high concentration of the BMP can specify epidermal cells (54). Mouse ESs and hESCs have been shown to differentiate into keratinocytes when exposed to high concentrations of BMP (55). However, the efficiency of this differentiation was low due to suboptimal culture conditions. BMP signals can also enhance extra- embryonic differentiation (56), which explains the low efficiency of keratinocyte differentiation. Combined activation of BMP4 and retinoic acid (RA) results in higher keratinocyte production from hESCs (57). The exact mechanisms of RA and BMP signaling during keratinocyte differentiation are unclear, but some reports suggest RA (58) and BMP function to regulate p63 expression (59). P63 expression is essential for epithelial cell proliferation and development and RA positively regulates p63 expression in primary keratinocytes (60). Despite progress made in generating epidermal cells from pluripotent cells, there are shortcomings in shortening the duration of the process, maintaining the homogeneity of the cells and proliferation potency, and optimizing the culture condition of keratinocytes (61). 

### Extra-embryonic cells

Extra-embryonic cells are defined as cells that do not form any of the embryo’s tissues, but are essential for its development. They give rise to the placenta, amniotic and chorionic membranes that supply the nutrients and oxygen, and safeguard the embryo from environmental stresses. Successful differentiation of hESCs into placenta cells has been initially described by Xu et al. (56), where they administered BMP4 *in vitro*. As previously described, FGF and TGFβ signals interplay to specify a hESC destiny towards the mesendoderm (6). Inhibition of the ERK pathway downstream of FGF and blockage of Activin receptors leads to BMP mediated extra- embryonic differentiation (Fig .2C) (6). Unlike the mesendoderm-inducing signals, which require high levels of ERK activation, placenta cells are generated in the absence of external FGF signaling, without blocking internal signals (6). These extra-embryonic cells have the capacity to respond to FGF signals and dedifferentiate to mesodermal cells (62). 

## Conclusion

Pluripotent cells have enhanced our understanding of developmental biology and they hold potential in cell-replacement therapies. Therefore, robust and efficient protocols are needed for direct differentiation of pluripotent cells into the desired cell types, with minimal contamination. The current review has focused on external and internal signals essential for maintaining the pluripotency of hPSCs and early differentiation signals. Despite progress made in dissecting the early signals that affect hPSCs, there remains a gap in our understanding of late signaling, how organs develop *in vivo*, and which signals correspond to further differentiation of each embryonic layer *in vitro*. The effects of the niche (matrix and cell-cell interactions) on these signals are unknown. The lack of knowledge regarding niche-specific effects on the behavior of stem cells and how these signals control cellular function remain to be illuminated. 

## References

[1] Bendall SC, Stewart MH, Menendez P, George D, Vijayaragavan K, Werbowetski-Ogilvie T (2007). IGF and FGF cooperatively establish the regulatory stem cell niche of pluripotent human cells in vitro. Nature.

[2] Mason I (2007). Initiation to end point: the multiple roles of fibroblast growth factors in neural development. Nat Rev Neurosci.

[3] Zhou J, Su P, Wang L, Chen J, Zimmermann M, Genbacev O (2009). mTOR supports long-term self-renewal and suppresses mesoderm and endoderm activities of human embryonic stem cells. Proc Natl Acad Sci USA.

[4] Singh AM, Reynolds D, Cliff T, Ohtsuka S, Mattheyses AL, Sun Y (2012). Signaling network crosstalk in human pluripotent cells: a Smad2/3-regulated switch that controls the balance between self-renewal and differentiation. Cell Stem Cell.

[5] Greber B, Wu G, Bernemann C, Joo JY, Han DW, Ko K (2010). Conserved and divergent roles of FGF signaling in mouse epiblast stem cells and human embryonic stem cells. Cell Stem Cell.

[6] Yu P, Pan G, Yu J, Thomson JA (2011). FGF2 sustains NANOG and switches the outcome of BMP4-induced human embryonic stem cell differentiation. Cell Stem Cell.

[7] Rommel C, Clarke BA, Zimmermann S, Nuñez L, Rossman R, Reid K (1999). Differentiation stage-specific inhibition of the Raf-MEK-ERK pathway by Akt. Science.

[8] Kunath T, Saba-El-Leil MK, Almousailleakh M, Wray J, Meloche S, Smith A (2007). FGF stimulation of the Erk1/2 signalling cascade triggers transition of pluripotent embryonic stem cells from self-renewal to lineage commitment. Development.

[9] Stavridis MP, Lunn JS, Collins BJ, Storey KG (2007). A discrete period of FGF-induced Erk1/2 signalling is required for vertebrate neural specification. Development.

[10] Besser D (2004). Expression of nodal, lefty-a, and lefty-B in undifferentiated human embryonic stem cells requires activation of Smad2/3. J Biol Chem.

[11] Vallier L, Alexander M, Pedersen RA (2005). Activin/Nodal and FGF pathways cooperate to maintain pluripotency of human embryonic stem cells. J Cell Sci.

[12] Vallier L, Mendjan S, Brown S, Chng Z, Teo A, Smithers LE (2009). Activin/Nodal signalling maintains pluripotency by controlling Nanog expression. Development.

[13] Xu RH, Sampsell-Barron TL, Gu F, Root S, Peck RM, Pan G (2008). NANOG is a direct target of TGFbeta/activin-mediated SMAD signaling in human ESCs. Cell Stem Cell.

[14] Boyer LA, Lee TI, Cole MF, Johnstone SE, Levine SS, Zucker JP (2005). Core transcriptional regulatory circuitry in human embryonic stem cells. Cell.

[15] Chng Z, Teo A, Pedersen RA, Vallier L (2010). SIP1 mediates cell-fate decisions between neuroectoderm and mesendoderm in human pluripotent stem cells. Cell Stem Cell.

[16] Hassani SN, Totonchi M, Sharifi-Zarchi A, Mollamohammadi S, Pakzad M, Moradi S (2014). Inhibition of TGFβ signaling promotes ground state pluripotency. Stem Cell Rev.

[17] Hassani SN, Pakzad M, Asgari B, Taei A, Baharvand H (2014). Suppression of transforming growth factor β signaling promotes ground state pluripotency from single blastomeres. Hum Reprod.

[18] Komiya Y, Habas R (2008). Wnt signal transduction pathways. Organogenesis.

[19] Taei A, Hassani SN, Eftekhari-Yazdi P, Rezazadeh Valojerdi M, Nokhbatolfoghahai M, Masoudi NS (2013). Enhanced generation of human embryonic stem cells from single blastomeres of fair and poor-quality cleavage embryos via inhibition of glycogen synthase kinase β and Rho-associated kinase signaling. Hum Reprod.

[20] Sumi T, Tsuneyoshi N, Nakatsuji N, Suemori H (2008). Defining early lineage specification of human embryonic stem cells by the orchestrated balance of canonical Wnt/beta-catenin, Activin/Nodal and BMP signaling. Development.

[21] Bain J, Plater L, Elliott M, Shpiro N, Hastie CJ, McLauchlan H (2007). The selectivity of protein kinase inhibitors: a further update. Biochem J.

[22] Merrill BJ (2012). Wnt pathway regulation of embryonic stem cell self-renewal. Cold Spring Harb Perspect Biol.

[23] Blauwkamp TA, Nigam S, Ardehali R, Weissman IL, Nusse R (2012). Endogenous Wnt signalling in human embryonic stem cells generates an equilibrium of distinct lineage-specified progenitors. Nat Commun.

[24] Tada S, Era T, Furusawa C, Sakurai H, Nishikawa S, Kinoshita M (2005). Characterization of mesendoderm: a diverging point of the definitive endoderm and mesoderm in embryonic stem cell differentiation culture. Development.

[25] Oshimori N, Fuchs E (2012). The harmonies played by TGF-β in stem cell biology. Cell Stem Cell.

[26] Teo AK, Ali Y, Wong KY, Chipperfield H, Sadasivam A, Poobalan Y (2012). Activin and BMP4 synergistically promote formation of definitive endoderm in human embryonic stem cells. Stem Cells.

[27] Greber B, Coulon P, Zhang M, Moritz S, Frank S, Muller- Molina AJ (2011). FGF signalling inhibits neural induction in human embryonic stem cells. EMBO J.

[28] Barnes RM, Firulli BA, Conway SJ, Vincentz JW, Firulli AB (2010). Analysis of the Hand1 cell lineage reveals novel contributions to cardiovascular, neural crest, extra-embryonic, and lateral mesoderm derivatives. Dev Dyn.

[29] Sapkota G, Alarcón C, Spagnoli FM, Brivanlou AH, Massagué J (2007). Balancing BMP signaling through integrated inputs into the Smad1 linker. Mol Cell.

[30] Sasai Y, Lu B, Steinbeisser H, De Robertis EM (1995). Regulation of neural induction by the Chd and Bmp-4 antagonistic patterning signals in Xenopus. Nature.

[31] Tropepe V, Hitoshi S, Sirard C, Mak TW, Rossant J, van der Kooy D (2001). Direct neural fate specification from embryonic stem cells: a primitive mammalian neural stem cell stage acquired through a default mechanism. Neuron.

[32] Hemmati-Brivanlou A, Melton D (1997). Vertebrate neural induction. Annu Rev Neurosci.

[33] Chambers SM, Fasano CA, Papapetrou EP, Tomishima M, Sadelain M, Studer L (2009). Highly efficient neural conversion of human ES and iPS cells by dual inhibition of SMAD signaling. Nat Biotechnol.

[34] Smith JR, Vallier L, Lupo G, Alexander M, Harris WA, Pedersen RA (2008). Inhibition of Activin/Nodal signaling promotes specification of human embryonic stem cells into neuroectoderm. Dev Biol.

[35] Thisse C, Thisse B (1999). Antivin, a novel and divergent member of the TGFbeta superfamily, negatively regulates mesoderm induction. Development.

[36] Verschueren K, Remacle JE, Collart C, Kraft H, Baker BS, Tylzanowski P (1999). SIP1, a novel zinc finger/homeodomain repressor, interacts with Smad proteins and binds to 5'-CACCT sequences in candidate target genes. J Biol Chem.

[37] Delalande JM, Guyote ME, Smith CM, Shepherd IT (2008). Zebrafish sip1a and sip1b are essential for normal axial and neural patterning. Dev Dyn.

[38] Miyoshi T, Maruhashi M, Van De Putte T, Kondoh H, Huylebroeck D, Higashi Y (2006). Complementary expression pattern of Zfhx1 genes Sip1 and deltaEF1 in the mouse embryo and their genetic interaction revealed by compound mutants. Dev Dyn.

[39] Wang Z, Oron E, Nelson B, Razis S, Ivanova N (2012). Distinct lineage specification roles for NANOG, OCT4, and SOX2 in human embryonic stem cells. Cell Stem Cell.

[40] Garnett AT, Square TA, Medeiros DM (2012). BMP, Wnt and FGF signals are integrated through evolutionarily conserved enhancers to achieve robust expression of Pax3 and Zic genes at the zebrafish neural plate border. Development.

[41] Marchal L, Luxardi G, Thomé V, Kodjabachian L (2009). BMP inhibition initiates neural induction via FGF signaling and Zic genes. Proc Natl Acad Sci USA.

[42] Yoo YD, Huang CT, Zhang X, Lavaute TM, Zhang SC (2011). Fibroblast growth factor regulates human neuroectoderm specification through ERK1/2-PARP-1 pathway. Stem Cells.

[43] LaVaute TM, Yoo YD, Pankratz MT, Weick JP, Gerstner JR, Zhang SC (2009). Regulation of neural specification from human embryonic stem cells by BMP and FGF. Stem Cells.

[44] Tesar PJ, Chenoweth JG, Brook FA, Davies TJ, Evans EP, Mack DL (2007). New cell lines from mouse epiblast share defining features with human embryonic stem cells. Nature.

[45] Nie X, Wang Q, Jiao K (2011). Dicer activity in neural crest cells is essential for craniofacial organogenesis and pharyngeal arch artery morphogenesis. Mech Dev.

[46] García-Castro MI, Marcelle C, Bronner-Fraser M (2002). Ectodermal Wnt function as a neural crest inducer. Science.

[47] Mayor R, Guerrero N, Martínez C (1997). Role of FGF and noggin in neural crest induction. Dev Biol.

[48] Marchant L, Linker C, Ruiz P, Guerrero N, Mayor R (1998). The inductive properties of mesoderm suggest that the neural crest cells are specified by a BMP gradient. Dev Biol.

[49] Wawersik S, Evola C, Whitman M (2005). Conditional BMP inhibition in Xenopus reveals stage-specific roles for BMPs in neural and neural crest induction. Dev Biol.

[50] Steventon B, Araya C, Linker C, Kuriyama S, Mayor R (2009). Differential requirements of BMP and Wnt signalling during gastrulation and neurulation define two steps in neural crest induction. Development.

[51] Sauka-Spengler T, Bronner-Fraser M (2008). A gene regulatory network orchestrates neural crest formation. Nat Rev Mol Cell Biol.

[52] Menendez L, Yatskievych TA, Antin PB, Dalton S (2011). Wnt signaling and a Smad pathway blockade direct the differentiation of human pluripotent stem cells to multipotent neural crest cells. Proc Natl Acad Sci USA.

[53] Chen J, Yan H, Ren DN, Yin Y, Li Z, He Q (2014). LRP6 dimerization through its LDLR domain is required for robust canonical Wnt pathway activation. Cell Signal.

[54] Wilson PA, Hemmati-Brivanlou A (1995). Induction of epidermis and inhibition of neural fate by Bmp-4. Nature.

[55] Green H, Easley K, Iuchi S (2003). Marker succession during the development of keratinocytes from cultured human embryonic stem cells. Proc Natl Acad Sci USA.

[56] Xu RH, Chen X, Li DS, Li R, Addicks GC, Glennon C (2002). BMP4 initiates human embryonic stem cell differentiation to trophoblast. Nat Biotechnol.

[57] Metallo CM, Ji L, de Pablo JJ, Palecek SP (2008). Retinoic acid and bone morphogenetic protein signaling synergize to efficiently direct epithelial differentiation of human embryonic stem cells. Stem Cells.

[58] Chen CF, Lohnes D (2005). Dominant-negative retinoic acid receptors elicit epidermal defects through a non-canonical pathway. J Biol Chem.

[59] Bakkers J, Hild M, Kramer C, Furutani-Seiki M, Hammerschmidt M (2002). Zebrafish DeltaNp63 is a direct target of Bmp signaling and encodes a transcriptional repressor blocking neural specification in the ventral ectoderm. Dev Cell.

[60] Senoo M, Pinto F, Crum CP, McKeon F (2007). p63 Is essential for the proliferative potential of stem cells in stratified epithelia. Cell.

[61] Liu S, Zhang H, Duan E (2013). Epidermal development in mammals: key regulators, signals from beneath, and stem cells. Int J Mol Sci.

[62] Das P, Ezashi T, Schulz LC, Westfall SD, Livingston KA, Roberts RM (2007). Effects of fgf2 and oxygen in the bmp4-driven differentiation of trophoblast from human embryonic stem cells. Stem Cell Res.

